# Ever-increasing Caesarean section and its economic burden in Bangladesh

**DOI:** 10.1371/journal.pone.0208623

**Published:** 2018-12-10

**Authors:** Mohammad Rifat Haider, Mohammad Masudur Rahman, Md. Moinuddin, Ahmed Ehsanur Rahman, Shakil Ahmed, M. Mahmud Khan

**Affiliations:** 1 Department of Health Promotion, Education and Behavior, Arnold School of Public Health, University of South Carolina, Columbia, SC, United States of America; 2 Department of Health Services Policy and Management, Arnold School of Public Health, University of South Carolina, Columbia, SC, United States of America; 3 Department of Public Health and Informatics, Jahangirnagar University, Savar, Dhaka, Bangladesh; 4 Maternal and Child Health Division (MCHD), International Centre for Diarrhoeal Disease Research, Bangladesh (icddr,b), Dhaka, Bangladesh; 5 The World Bank, Dhaka, Bangladesh; 6 Department of Statistical Science, University of Padova, Padova, Italy; Liverpool School of Tropical Medicine, UNITED KINGDOM

## Abstract

**Background:**

Cesarean Section (CS) delivery has been increasing rapidly worldwide and Bangladesh is no exception. In Bangladesh, the CS rate has increased from about 3% in 2000 to about 24% in 2014. This study examines trend in CS in Bangladesh over the last fifteen years and implications of this increasing CS rates on health care expenditures.

**Methods:**

Birth data from Bangladesh Demographic and Health Survey (BDHS) for the years 2000–2014 have been used for the trend analysis and 2010 Bangladesh Maternal Mortality Survey (BMMS) data were used for estimating health care expenditure associated with CS.

**Results:**

Although the share of institutional deliveries increased four times over the years 2000 to 2014, the CS deliveries increased eightfold. In 2000, only 33% of institutional deliveries were conducted through CS and the rate increased to 63% in 2014. Average medical care expenditure for a CS delivery in Bangladesh was about BDT 22,085 (USD 276) in 2010 while the cost of a normal delivery was BDT 3,565 (USD 45). Health care expenditure due to CS deliveries accounted for about 66.5% of total expenditure on all deliveries in Bangladesh in 2010. About 10.3% of Total Health Expenditure (THE) in 2010 was due to delivery costs, while CS costs contribute to 6.9% of THE and rapid increase in CS deliveries will mean that delivering babies will represent even a higher proportion of THE in the future despite declining crude birth rate.

**Conclusion:**

High CS delivery rate and the negative health outcomes associated with the procedure on mothers and child births incur huge economic burden on the families. This is creating inappropriate allocation of scarce resources in the poor economy like Bangladesh. Therefore it is important to control this unnecessary CS practices by the health providers by introducing litigation and special guidelines in the health policy.

## Background

The rate of Caesarean Section (CS) in Bangladesh has increased alarmingly during the last two decades. In 2000, CS rate was only 3% and it increased to 24% in 2014 [1, 2]. Although this is not the highest CS rate in the Asian region, the growth rate of CS in Bangladesh is alarming. A number of factors are likely to be associated with increasing CS including higher prevalence of medical indications for CS (maternal age, obesity, multiple gestation, diabetes, and hypertension/preeclampsia), increased access to modern health care services, improved economic status of population, changes in cultural and social factors and supply induced demand for CS [3–6].

CS rates in Bangladesh exceed those for other similar developing countries and it is unclear to what extent the adoption of CS is based on strict clinical indications and guidelines [7, 8]. CS is an important, potentially lifesaving intervention if it is conducted for cases with clear indications of CS [9, 10]. Elective CS should not be encouraged because cesarean delivery is a major abdominal surgery and significantly riskier than normal vaginal delivery. CS is associated with higher rates of infection, injury, short-term and long-term disability. It may also delay initiation of breastfeeding and in some cases, it may require emergency hysterectomy [9–11]. Many CS cases create persistent pain and may complicate future deliveries [12, 13]. One of the main reasons to perform CS is to improve neonatal outcomes and reduce risk of mortality and morbidity. However, evidence suggests CS is associated with a greater risk of respiratory distress, asphyxia, and other pulmonary infections in infants [7, 14, 15]. In some settings it was observed that maternal mortality was greater for CS than for vaginal birth, probably because of adoption of unnecessary CS [16].

There is little or no evidence that community-level CS rate has any significant maternal and perinatal benefits when it exceeds an upper threshold level. In fact, some studies have shown a link between high rate of CS and poor health outcomes [17, 18]. For Bangladesh as well, rapidly increasing CS is not associated with declining maternal mortality ratio (MMR). Recent Bangladesh Maternal Mortality Survey (BMMS) found the MMR to be stagnant, 192 in 2010 and 194 (per 100,000 live births) in 2016 [16, 19].

Sometimes social pressures and other non-clinical factors play significant role on the choice of CS delivery by women [20]. This is more pronounced in developing countries where quality of care is extremely poor and adverse outcomes of medical interventions are common. Studies in Brazil showed that women were encouraged by the social attitudes towards avoidance of labor pain to undergo CS [21, 22]. Other studies have shown that doctors tend to recommend CS by taking advantage of women’s concerns over potential complications arising from childbirth [21].

A number of risk factors are associated with the adoption of CS to preserve health and wellbeing of the mother and the baby. These factors include maternal age, maternal weight, parity, prolonged labor, mother with HIV infection, previous CS delivery, dystocia, breech presentation, placenta previa, and suspected fetal complications [23–26]. Therefore, it is important to control for these individual level risk factors in explaining the trend in CS.

Another important aspect to consider is the financial incentives associated with CS delivery from the point of view of health care providers. Net income of health care providers is higher for CS delivery compared to vaginal delivery [27, 28]. In a report it was found that the cost of a normal delivery at a hospital in poor countries of Africa and Latin America ranges from US$ 10–35 and a caesarean section or a complicated vaginal delivery can cost from US$ 50–100 [29]. In addition, time spent by physicians on delivery of babies and/or degree of uncertainty about the exact time of delivery also play some role in deciding whether to adopt CS or not for delivery. CS takes less time for obstetricians and gynecologists unlike the normal childbirth and the surgery can be scheduled in advance lowering uncertainty about exact time of delivery [27].

World Health Organization in the World Health Report 2010 estimated that 6 million unnecessary CS were performed in 2008 and the cost of this unnecessary CS was about US$ 2.32 billion [30, 31]. Increasing the income of the health care providers may be one of the reasons for conducting unnecessary CS deliveries [32]. Unnecessary CS not only have adverse health effects but also impose significantly higher financial burden on women [33, 34]. Compared to normal delivery, CS requires higher days of hospital stay implying higher medical care cost and loss of income for the household. If CS takes place at private health care institutions, medical care expenses are usually even higher [35].

Considering the potential negative consequences of CS when conducted without any clinical indications, this paper aims to examine the trends of CS in Bangladesh over the last fifteen years. The study will also estimate average and total costs of CS in Bangladesh and the financial implications of CS at the individual and national level.

## Methods

### Data source

This study has used two different sets of data to analyze the trend of CS and cost of CS in Bangladesh. The trend in CS has been estimated using Bangladesh Demographic and Health Survey (BDHS) data sets of 2000, 2004, 2007, 2011 and 2014. Birth data from the 2010 BMMS have also been used to obtain information on costs associated with CS for this study. BMMS is a nationally representative survey which was conducted for the second time in 2010 after the first survey conducted in 2001. The BDHS is also a nationally representative survey that follows a multi-stage cluster sampling technique. Information on women who gave birth in three years preceding the survey was used in this study (N = 4,627). This study was exempt from the ethical review approval because it uses publicly available de-identified data.

### Data analysis

To examine the trend in CS in Bangladesh, five cycles of BDHS data were used. The BDHS data sets used were collected in 2000, 2004, 2007, 2011, and 2014. Three different indicators were analyzed for understanding the trend in delivery and delivery methods. The first measure is the number of deliveries performed in health facilities as percentage of all deliveries and the second measure examines CS deliveries as a percentage of total deliveries. The final measure is the CS deliveries as percent of all institutional deliveries.

We used the 2010 BMMS data for estimating the costs of delivery, e.g., both normal delivery and CS; because in BMMS survey information on delivery costs were collected but BDHS does not have any information on costs. Total cost for delivery includes costs incurred during pregnancy, delivery and after delivery for transport, medicine, hospital services, and other. We conducted descriptive analysis to have a view of the characteristics of the study participants as well as those who gave birth to their child by CS in preceding three years of the survey. Bivariate analysis was also done among two types of deliveries. Chi-square test was performed to see any significant difference among the categories of each variable with the dependent variable of nature of delivery.

Multivariate analysis was done using a generalized linear model with gamma family and log link with the outcome variable being delivery costs. Modified Park test, Pregibon link test and Pearson correlation test were used to determine the link. All analyses were performed using Stata 14.2 [36]. After fitting the regression model, the cost of CS as well as normal delivery in Bangladesh were predicted based on the coefficient values of the model. Average predicted costs for two types of deliveries were calculated. Using the rate of CS from BDHS 2014 and the number of birth in Bangladesh in 2014 from UN population division estimates, we calculated the total number and costs of CS and normal delivery in Bangladesh. We used the Bangladesh National Health Accounts 1997–2012 for calculating Total Health Expenditure (THE) for 2014 (Ref.). Using the consumer price index collected from World Bank data (https://data.worldbank.org/indicator/FP.CPI.TOTL?locations=BD) [37], we converted the THE of 2012 to 2014 level. Finally, we estimated the costs of the institutional delivery as a fraction of THE [38].

## Results

### Trends in CS in Bangladesh

**Fig 1:**
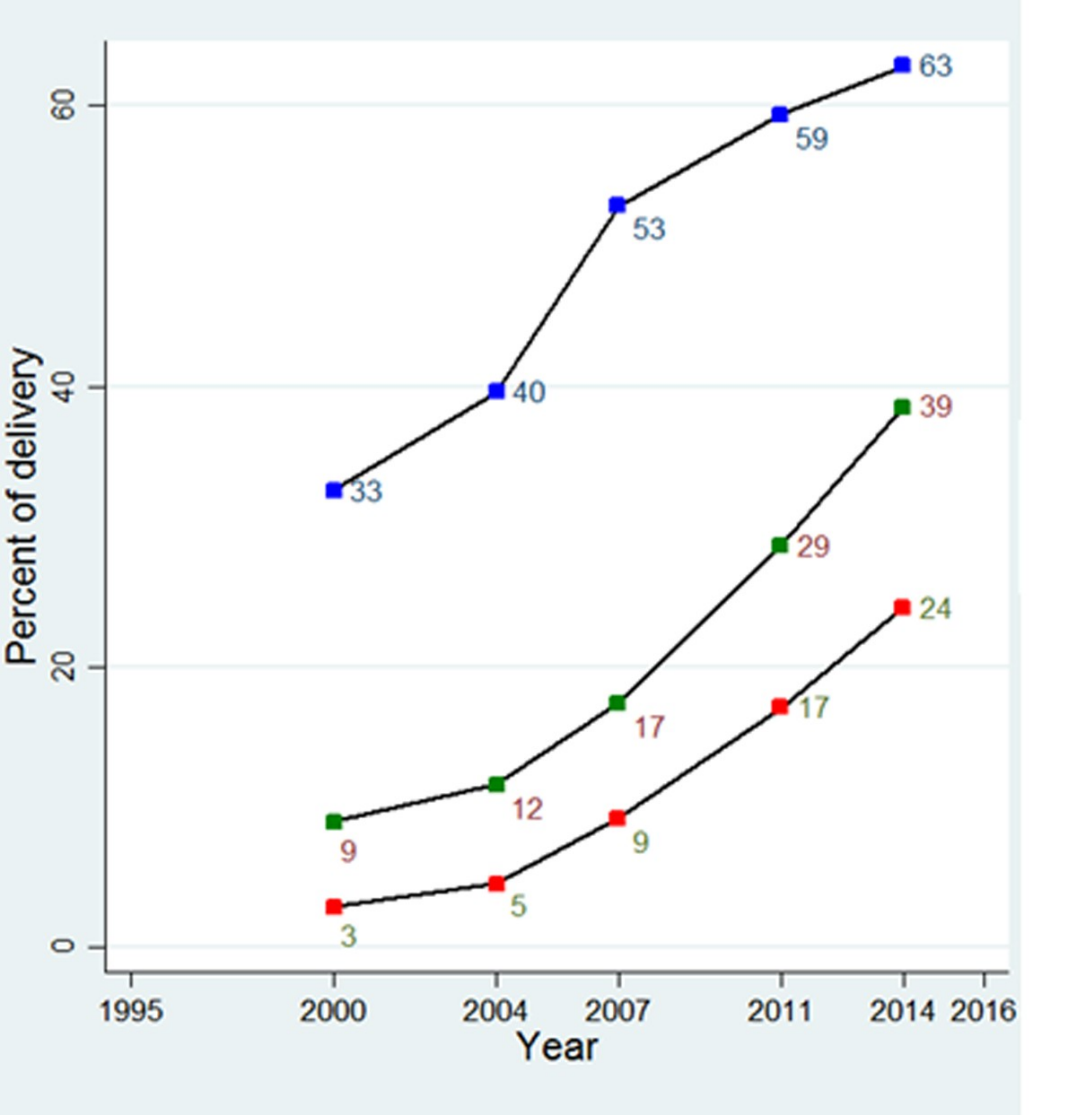
Trends in delivery by caesarian section in Bangladesh, 2000 to 2014. “Designated with a blue square” CS among facility delivery. “Designated with a green square” Facility delivery among all delivery. “Designated with a red square” CS among all delivery.

Fig 1 shows the trend in delivery by CS in Bangladesh over the years 2000 to 2014. One principal concern among health policy makers and practitioners in Bangladesh is that even in 2014 most of the deliveries (61%) were performed at home. The share of institutional deliveries increased four times over the fifteen years from 9% to 39%. At the same time, CS has increased from only 3% of all deliveries in 2000 to 24% in 2014. Since all CS takes place in health facilities, continued increase in the use of health facilities for deliveries and CS rate among institutional deliveries will indicate how the CS is likely to change in the future. Fig 1 shows that in 2000 only 33% of institutional deliveries were CS and the rate almost doubled to 63% in 2014.

### Characteristics of the women under study and who had CS

Table 1 shows the descriptive statistics and the proportion of CS by different characteristics of the women and the households. Out of 18,733 women in the survey in 2010, 3,323 (11.9%) experienced CS in Bangladesh. While most of the women belonged to 20–24 year (48.6%) and 13–19 year (40.6%) age brackets, there was no significant difference in CS rate across different age groups. More than half of the women had secondary or higher education (62.4%), while the adoption of CS is higher among the secondary or higher educated women (19.4%). While most of the respondents lived in rural areas (74.0%), almost one-fifths (18.6%) of the urban women underwent CS. Rate of CS did not affect much by the wealth quintile differences where the rate of CS for poorest quintile was 11.6% and rate of the richest quintile was 10.8%. Most of the women (62.6%) received three or less antenatal care visits, while 26.2% women who had CS had four or more ANC visits. While 42.6% of the women had delivery in private facilities, almost one-fifth of those who had delivery in private facilities had CS.

**Table 1:** Survey weighted characteristics of the respondents and those who delivered by CS, BMMS 2010 (N = 18,733 and 3,323).

Characteristics	Number of respondents	% delivered by C-Section	*p-value (difference in proportions)*
	(%) N	(%) N	
**Total**	100.0 (18,733)	11.9 (3,323)	
**Maternal age (years)**			
13–19	40.6 (7,421)	9.8 (315)	0.000
20–24	48.6 (9,186)	12.2 (1,190)	
25–29	9.02 (1,751)	11.3 (975)	
30–34	1.52 (317)	10.5 (540)	
35–39	0.20 (49)	9.6 (235)	
40–49	0.04 (9)	5.6 (68)	
**Maternal Education (levels of schooling)**			
No education	9.7 (1,876)	3.0 (223)	0.000
Incomplete Primary	11.9 (2,217)	5.2 (234)	
Completed Primary	16.0 (2,890)	6.7 (312)	
Secondary or Higher	62.4 (11,750)	19.4 (2,554)	
**Religion**			
Other	9.8 (2,087)	16.3 (452)	0.000
Islam	90.2 (16,646)	10.5 (2,871)	
**Residence**			
Rural	74.0 (10,610)	8.6 (1,394)	0.000
Urban	26.0 (8,123)	18.6 (1,929)	
**Wealth Index**			
Poorest	20.6 (3,861)	11.6 (706)	0.626
Poorer	18.9 (3,593)	11.1 (638)	
Middle	19.4 (3,576)	10.6 (638)	
Richer	19.2 (3,610)	10.7 (607)	
Richest	21.9 (4,093)	10.8 (734)	
Number of antenatal care visits recieved*			
1–3	62.6 (3,151)	10.3 (1,411)	0.000
4+	37.4 (2,042)	26.2 (1,733)	
Antenatal care place**			
Home	11.2 (525)	4.3 (100)	0.000
Public Facilities	15.1 (853)	8.8 (306)	
NGO Facilities	31.1 (1,592)	21.0 (1,215)	
Private and Other Facilities	42.6 (2,202)	17.2 (1,509)	
**Delivery Assistance**			
None	0.3 (18)	0 (0)	0.000
Health Professional	39.0 (2,679)	44.3 (3,321)	
Other	60.6 (3,747)	0.01 (2)	
**Delivery Place**			
Home	65.4 (4,093)	0 (0)	0.000
Public Facilities	14.5 (1,025)	34.3 (982)	
NGO Facilities	16.6 (1,094)	31.6 (178)	
Private and Other Facilities	3.6 (235)	68.4 (2,163)	

*N = 19,142

**N = 19,036

There has been a significant increase in institutional delivery and CS rates in Bangladesh during the last two decades. However, the rate of increase was higher for CS than for institutional deliveries. The institutional delivery rate increased from 9% during 2000 to only 39% in 2014. With the current rate of increase, it will be difficult for Bangladesh to achieve its national target of 70% institutional delivery by 2025. Meanwhile, the population-based CS rate increased dramatically from 3% in 2000 to 24% in 2014. The annual rate of increase is 1.5%. If this rate continues in the same way the CS rate will reach to 40.5% among all deliveries by the year 2025. Given the rapid expansion of both private and public facilities and the growing trend of CS in the country the CS rate will reach that level if not more. For a resource poor country like Bangladesh this is really a grave concern.

### Delivery costs drivers in Bangladesh

Table 2 shows the drivers of delivery costs in Bangladesh. With increasing age of the mother delivery costs also increased. Similarly, with higher educational attainment of the mother delivery costs also increased. There was no significant effect of residence and wealth index on delivery costs. Facility delivery, be it CS or normal vaginal delivery, increased the costs, while four or more ANC visits and ANC visits in facilities increased the delivery costs. Type of delivery assistants had no effect on the costs of delivery.

**Table 2:** Survey weighted multivariable generalized linear model results with log link and gamma family for delivery costs in Bangladesh (N = 17,442), BMMS 2010.

Variables	Coefficient	Std. Error	*p-value*
**CS**			
No	Ref.		
Yes	0.78	0.08	0.00
**Maternal age (years)**			
13–19	Ref.		
20–24	0.11	0.05	0.01
25–29	0.27	0.07	0.00
30–34	0.26	0.12	0.03
35–39	0.89	0.49	0.07
40–49	0.57	0.10	0.00
**Maternal Education (levels of schooling)**			
No education	Ref.	-	-
Incomplete Primary	0.23	0.10	0.03
Completed Primary	0.16	0.09	0.07
Secondary or Higher	0.36	0.08	0.00
**Religion**			
Other	Ref.	-	-
Islam	0.12	0.06	0.04
**Residence**			
Rural	Ref.	-	-
Urban	0.04	0.04	0.42
**Wealth Index**			
Poorest	Ref.	-	-
Poorer	-0.03	0.06	0.57
Middle	-0.00	0.06	0.98
Richer	0.08	0.07	0.22
Richest	0.02	0.06	0.66
**CS**			
No	Ref.		
Yes	0.79	0.08	0.00
**Number of antenatal care visits recieved**			
1–3	Ref.	-	-
4+	0.21	0.04	0.00
**Antenatal care place**			
Home	Ref.	-	-
Public Facilities	0.40	0.08	0.00
NGO Facilities	0.71	0.07	0.00
Private and Other Facilities	0.72	0.07	0.00
**Delivery Assistance**			
None	Ref.	-	-
Health Professional	0.48	0.58	0.40
Other	-0.17	0.57	0.77
**Delivery Place**			
Home	Ref.		
Public Facilities	0.29	0.08	0.00
NGO Facilities	0.44	0.13	0.00
Private and Other Facilities	0.85	0.13	0.00
**Constant**	6.63	0.58	0.00

### Costs of CS and its contribution to total health expenditure in Bangladesh

Using the BMMS 2010 data we found that for total cost of a CS on average was BDT 22,085, while the cost for normal delivery was on average BDT 3,565. We estimated the total number of births (3,561,124) in 2014 from UN Population division data and applying the rate of CS from BDHS 2014 data we have got that total number of CS in 2014 was 858,160, while other 2,673,361 babies were delivered by normal delivery. Total cost of CS in 2014 was BDT 18,952 million, whereas the total cost for normal delivery was BDT 9,531 million. In 2012, National Total Health Expenditure (THE) was BDT 325,094 million. Using CPI from World Bank data, THE in 2014 would be BDT 374,012 million (Table 3). In 2014, the total delivery cost of BDT 38,533 million was 10.3% of the THE. Costs for CS contribute 66.5% of the total delivery cost in 2014, which is 6.9% of the THE.

**Table 3:** Cost of delivery by place of delivery (last birth in three years preceding the survey).

Type of delivery	Average cost per delivery	Total birth in 2014	Total cost for delivery	Total cost for delivery	Total cost for delivery
	Mean (SD)	(Source: BDHS 2014)	2010 BDT (in millions)	2014 BDT (in millions)	2014 USD (in millions)
Caesarian Section	22,085 (8,045)	858,160	18,952 Million	25,639 Million	320 Million
Normal Delivery	3,565 (2,918)	2,673,361	9,531 Million	12,893 Million	161 Million
Total	6,349 (16,079)	3,531,521	28,483 Million	38,533 Million	482 Million

## Discussion

The study aimed to explore the trends of CS as delivery procedure in Bangladesh between 2000 and 2014 and described the cost implication of CS at individual and national level. It has been observed that facility delivery increased significantly but CS practically doubled to the rate at which facility delivery increased over the last fifteen years in Bangladesh.

According to the study findings the majority of CS is performed at NGO and private facilities in Bangladesh. Educated and younger mothers tend to undergo CS more than normal vaginal delivery. It is reported that the effect of the mother’s educational level operates through age and parity [39]. The CS rates among different socioeconomic groups are almost similar in this study though in many cases it was found that the CS rates are the combination of low service utilization (CS) by the poorest segments and higher service utilization by the richest segments of the population [40]. This may be due to the fact that number of private NGOs and clinics are rising and the services for maternal and child care have now become more available and accessible by general population of the country [28]. A study in China showed that since the 1990s, the country has seen a dramatic increase in the rates of caesarean delivery and the structures of the healthcare system including the private hospitals, NGOs and clinics have contributed to this current caesarean rate. In another study in Brazil, women, especially those who deliver in private hospitals the cesarean rates is in the range of 80–90% due to rapid increase of private hospitals and clinics [20, 21]. Thus our study findings are consistent with other studies.

Whether the increasing trend of CS rates reflect actual gains towards meeting the need for obstetric care for the country is difficult to tell. However, it can be said fairly that all-cause CS may comprise women who actually require a surgical intervention in order to save their or their baby's life as well as women for whom there is no clinical requirement at all [41]. The increase in rates of CS observed over time in our study could not be fully explained by the increase in the rates of institutional birth alone. Additionally, it is likely to be driven by obstetricians’ preference to perform CS more than the vaginal delivery [28]. Also women’s demand for the procedure should be taken into consideration [42]. Like the increasing trend of CS rates, the number of clinical indications for a cesarean delivery has increased in recent years [43]. As a result, more attention has been paid to nonmedical determinants of a cesarean delivery [44]. In view of evidence for rising trends of CS deliveries in Bangladesh, concerns are raised about the risk of over-medicalizing birth and of the impact this has on scarce economic resources [20].

In Bangladesh, according to the study findings, almost one quarter of women give birth by CS where urban cesarean birth rates are much higher than rural rates. This finding is similar to other developing countries where CS rates also increasing in the urban areas where more educated and affluent people reside [20]. One important reason for this higher rate in urban areas may be due to presence of increased number of private hospitals, clinics and NGO based health facilities which have been established over the time [45]. Some other possible reasons for increasing CS rates that are reported in studies such as fear of labor pain, concerns about genital orientation after normal delivery, CS is safer for mother and the baby, the benefits and easy surgical procedure for the health professionals, prevention of any complications or outcomes of the baby. These findings are consistent with other studies [20, 46–48].

Increasing CS rates in Bangladesh presents problems at both individual and national level. Government health care expenditure in Bangladesh is one of the lowest in the world and there is no effective social health insurance policy currently available in the country, as a result some of the increase in demand for this procedure could be financed by patients. This increased cost most likely have taken place in new or expanded private hospitals. The structural changes in public and private health systems could partially explain the increasing CS rates. Between 1997 and 2014, the number of newly established general hospitals and clinics (both private and public) increased by more than 3 times (from 1286 in 1997 to 4317 in 2013) [45, 49].

The analysis also demonstrated that use of antenatal care is associated with a greater likelihood of CS. Frequent visit during the pregnancy period indicates the extent of use (medicalization) of antenatal care services by the mothers that could have influenced patient who prefers medical intervention, or may be acted as a type of medical behavior whereby doctors might be more inclined to perform CS. There are considerable differences in hospital types where CS is performed, delivery rates increased over the decade in the private hospitals more than to public hospitals [20].

Aside from the medical benefits and risks of CS for individual woman, an important consideration is the economic impact of this new rising trend [50]. According to Bangladesh Bureau of Statistics (BBS) average income of the people in Bangladesh is about USD 99 per month. However, performing each CS delivery was approximately USD 276 and for normal delivery the cost was around USD 45 [18, 51]. Therefore, in respect to household income, delivery costs are far beyond the means of most of the poor families. A study in Bangladesh estimated that 79% of households did not have enough money to pay for delivery and they had to borrow from friends and relatives [29, 52]. The average cost of a normal vaginal delivery (USD 45 or BDT 3,565) and a CS (USD 276 or BDT 22,085) in our study was substantially higher than other poor countries of Africa and Latin America but more comparable to results from regional studies like from India, Pakistan and Nepal [29]. Regional variations may be one of the reasons for this high expenditure in maternal health care particularly in case of CS in our study.

The rising trend in CS and high costs of the procedure incurred BDT 25,639 million (USD 320 million) which is almost 10.3% of the total health expenditure of the country reflects the huge healthcare market share of CS. If the unnecessary CS is curbed, then the poor families would not face catastrophic delivery costs and scarce healthcare money would be released for other more pressing health needs.

The CS rates in Bangladesh are consistently higher during the last decade what is considered beyond the justifiable range of 10% to 15% according to WHO standard [53]. Scientists, health care experts and policy makers have raised concern about this epidemic while the search for ideas and solutions to reduce unnecessary CS is on-going [54, 55]. However, reduction of unnecessary CS is not an inconsequential task and it will take significant time and efforts. To monitor CS rate, countries can adopt different policies and strategies. To keep the CS rate within expected level it is suggested that accreditation system of individual country should be strengthened, maintained and practiced [4]. Appropriate training, timely and regular supervision and leadership by senior physicians all are important in maintaining standards. It is recommended that the national societies of obstetrics and gynecology of the country should encourage the use of evidence-based protocols. Another important thing that is highly suggested is to perform routine clinical audits. It would be very useful to do routine clinical audits in facilities at all levels, which can be used to monitor the change of CS rate, improvement of practice and maintain a good quality of care [5, 6]. Given the limited resources of the country government of Bangladesh can focus on promotion of midwifery-based maternity care system and active involvement of new mothers in the development of local health services that emphasize birth as a normal process in order to content the rising trend of unnecessary CS. WHO also proposed the use of the Robson Classification system as a global standard for assessing and monitoring CS rates in 2015 [53, 56]. Government can incorporate this system in its health care policy.

Although this CS rate represents the country level data that may be useful for government and policy-makers to assess overall progress in maternal and infant health and to plan emergency obstetric care and resource utilization, this should be considered as mere averages and may conceal important inequalities within countries [40, 57]. In our previous paper we assessed the determinants of CS in Bangladesh [58] and more research is needed in future to explore the possible impact of different factors when considering potential interventions to reduce unnecessary CS and to further understand women’s health seeking behavior and the related social forces behind this increasing trend.

One of the limitations of the study is the recall period. In BDHS the recall period is five years and it is too long period to report all events related to CS births and its associated cost. There was no information in relation to CS as a necessary option or not or how clinical status of the patients might justify the operation. Further data on this could have strengthened the findings. Precise costing attributable to health care workers such as doctors and nurses in the hospital, for various activities related to CS (patient care, administrative work, personal time, opportunity cost, and laboratory cost) could not be performed as no information was available regarding these issues.

The strength of this paper is that it used recent data to show the rising rates of CS along with older nationally representative data to see the trend. It also used another nationally representative data (BMMS) for cost estimation. Nationally representative surveys such as the DHS are currently the most widely available source of population-based CS birth data. Moreover, the DHS data on CS are less likely to be influenced by reporting errors or bias, which is one of the main strengths in the analysis.
